# *Cask* methylation involved in the injury of insulin secretion function caused by interleukin1‐β

**DOI:** 10.1111/jcmm.16041

**Published:** 2020-11-14

**Authors:** Tian‐yuan Wang, Xing‐jing Liu, Jin‐yang Xie, Qing‐zhao Yuan, Yao Wang

**Affiliations:** ^1^ Department of Endocrinology Zhongda Hospital Institute of Diabetes School of Medicine Southeast University Nanjing China

**Keywords:** calcium/calmodulin‐dependent serine protein kinase, insulin secretion, interleukin1‐β, methyltransferases

## Abstract

Islet inflammation severely impairs pancreatic β‐cell function, but the specific mechanisms are still unclear. Interleukin1‐β (IL‐1β), an essential inflammatory factor, exerts a vital role in multiple physio‐pathologic processes, including diabetes. Calcium/calmodulin‐dependent serine protein kinase (CASK) is an important regulator especially in insulin secretion process. This study aims to unveil the function of CASK in IL‐1β–induced insulin secretion dysfunction and the possible mechanism thereof. Islets of Sprague‐Dawley (SD) rats and INS‐1 cells stimulated with IL‐1β were utilized as models of chronic inflammation. Insulin secretion function associated with *Cask* and DNA methyltransferases (DNMT) expression were assessed. The possible mechanisms of IL‐1β‐induced pancreatic β‐cell dysfunction were also explored. In this study, CASK overexpression effectively improved IL‐1β‐induced islet β‐cells dysfunction, increased insulin secretion. DNA methyltransferases and the level of methylation in the promoter region of *Cask* were elevated after IL‐1β administration. Methyltransferase inhibitor 5‐Aza‐2’‐deoxycytidine (5‐Aza‐dC) and si‐DNMTs partially up‐regulated CASK expression and reversed potassium stimulated insulin secretion (KSIS) and glucose‐stimulated insulin secretion (GSIS) function under IL‐1β treatment in INS‐1 and rat islets. These results reveal a previously unknown effect of IL‐1β on insulin secretion dysfunction and demonstrate a novel pathway for *Cask* silencing based on activation of DNA methyltransferases via inducible nitric oxide synthase (iNOS) and modification of gene promoter methylation.

## 1 INTRODUCTION

Islet inflammation is the hallmark of all types of diabetes mellitus (DM).^1^, ^2^ Accumulated evidence indicates that chronic islet inflammation exerts a strong role in pancreatic β‐cell dysfunction, including impaired insulin secretion function and diminished mass of islet β‐cells.^3^ IL‐1β has been identified as a main inflammatory mediator of eliciting islet β‐cell injury in diabetes,^4^ and animal studies and clinical trials blocking IL‐1β signalling pathway have proved to ameliorate β‐cell function and improve glucose homeostasis in DM,^5^, ^6^ yet the mechanism by which IL‐1β impairs islet β‐cell biology is not completely understood.

CASK is one of family members of membrane‐associated guanylate kinase (MAGUK) protein.^7^ It is a scaffolding protein that plays a vital role in exocytotic function of neurotransmitter granules in neurons.^8^, ^9^ Our previous work showed that CASK is critical in insulin secretion process in islet β‐cells, particularly in the part of insulin granules exocytosis.^10^, ^11^ CASK involves a process wherein FoxO1 mediated palmitate‐induced insulin secretion deficiency,^10^ and it has also been suggested that exendin‐4 induces insulin secretion via elevating CASK expression level in INS‐1 cells.^11^ CASK, as an important pseudokinase, is regulated by many transcript factors and modified by many modification modes, and participates in complex metabolic regulatory networks.^12^, ^13^, ^14^ However, the relationship between CASK and chronic islet inflammation has not been reported, and the exact mechanism and role of CASK are still unknown.

This study therefore aims to investigate whether the insulin secretion function under IL‐β exposure is related to the kinase function of CASK based on previous studies and goes on to explore the potential mechanism.

## 2 MATERIALS AND METHODS

### 2.1 Cell culture and treatment

The rat insulinoma cell line INS‐1 was cultured in RPMI‐1640 medium (Invitrogen) with 10% FBS, 50 μmol/L β‐mercaptoethanol (Sigma‐Aldrich), 100 U/mL penicillin and 100 μg/mL streptomycin. INS‐1 cells were grown at 37°C in a Thermo tissue‐culture incubator in a humidified atmosphere containing 5% CO_2_. The cells were seeded into 6‐well plates or 48‐well plates and incubated in modified medium with 0.5% (wt/vol.) BSA (Sigma‐Aldrich), treated with IL‐1β (Sigma‐Aldrich) at different concentrations (0.1, 1 or 10 ng/mL) and for different times (24, 48, or 72 hours), with or without 10 μmol/L of DNA methyltransferase inhibitor 5‐Aza‐dC (Sigma‐Aldrich). The culture medium was refreshed per 48 hours. The cells were collected for corresponding assays.

### 2.2 Cell transfection

The CASK overexpression plasmid, pEGFP‐N_2_‐CASK, has been constructed in previously research work.^10^ siRNA oligonucleotides against DNMT1, DNMT3a, DNMT3b or scrambled siRNA were synthesized and referred to previously work.^15^, ^16^ Fout thousand ng overexpression plasmid and 10 μL si‐RNA was transfected into INS‐1 cells in 3.5‐cm plates for 48 hours, with or without 1 ng/mL IL‐1β, using Lipofectamine 2000 (Invitrogen), following the instructions of manufacturer.

### 2.3 Islet isolation and culture

All animal experiments were approved by the Research Ethics Committee of Southeast University. 8‐week‐old SD rats and 18‐week‐old NOD/LtJ mice were obtained to use in this study. The animals were anaesthetized with pentobarbital natrium. And then the isolated islets were isolated and cultured as described previously.^17^ After equilibrating incubation for 3 hours, the islets were counted and seeded into 6‐well plates or 48‐well plates and treated according to the further experimental requirements. Islets were incubated in modified medium with 0.5% (wt/vol.) BSA, treated with IL‐1β at different concentrations (0.1, 1 or 10 ng/mL) and for different times (24, 48 or 72 hours), and 1 ng/mL IL‐1β combined with 10 μmol/L 5‐Aza‐dC treatment for 48 hours.

### 2.4 KSIS assay and GSIS assay

INS‐1 cells (7.0 × 10^4^ cells/well) and isolated rat islets (10 islets/well) were cultured in 48‐well plates with the corresponding treatments for GSIS and KSIS assays. INS‐1 cells or islets were pre‐incubated for 1 hour in HEPES‐balanced Krebs‐Ringer bicarbonate buffer (KRBH) containing 2.8 mmol/L glucose and 0.2% (wt/vol) BSA. The islets were incubated for 1 hour in KRBH containing low glucose (LG: 3.3 mmol/L) and high glucose (HG: 16.7 mmol/L). INS‐1 cells were incubated for 1 hour in KRBH containing basal glucose (LG: 2.8 mmol/L), stimulatory condition (HK: 50 mmol/L KCl and 2.8 mmol/L glucose). The supernatants were obtained, after the static incubation period. The insulin content was detected using radio‐immunoassay (RIA), as previously described.^18^

### 2.5 Measurement of cytosolic Ca^2＋^ concentration

For determination of intracellular Ca^2＋^ concentration, INS‐1 cells and rat islets were routinely plated on 48‐well plates and treated with IL‐1β according to the purpose of the experiment. Before Fura‐4/AM loading, cells were stabilized by a 20 minutes incubation in KRBH containing basal glucose before detecting. After the basic signal of the cells collecting, the buffer is immediately switching to HK (50 mmol/L KCl and 2.8 mmol/L glucose) or HG (16.7 mmol/L glucose). Results of Fura‐4/AM measurements were recorded at 510 nm. The relative Ca^2＋^concentration is plotted as the average ratio (HK/LG) of each experimental group ± SEM.

### 2.6 RNA extraction, reverse‐transcription polymerase chain reaction (RT‐PCR) and quantitative real‐time PCR (qPCR)

The INS‐1 cells were seeded in 3.5‐cm plates for different times and at different concentrations of IL‐1β or drugs. Total RNA was extracted using Trizol reagent (Invitrogen). Complementary DNA (cDNA) synthesis was performed using 500 ng total RNA and reversed by RT‐mix kit (Vazyme). The real‐time PCR with corresponding cDNA, primers and SYBR Green PCR mastermix (Vazyme) was performed using the LightCycler480 Sequence Detection System (Roche, Switzerland). All data were analysed using β‐actin as the reference. The sequences of primers are shown in (Table S1). The data were analysed by the 2^−ΔΔCt^ method.

### 2.7 Western blot analysis

INS‐1 cells were harvested with ice‐cold lysis buffer supplemented with complete proteinase inhibitor mixture (Roche). Other specific information of Western blotting assay was performed as previously described previously.^19^ The antibodies used are as follows: rabbit anti‐CASK antibody (1:1000; Cell Signaling), rabbit anti‐DNMT1 (1:1000; Cell Signaling), mouse anti‐DNMT3a (1:1000; Santa Cruz Biotechnology), rabbit anti‐DNMT3b (1:1000; Abcam), rabbit anti‐Akt(pan) antibody (1:1000; Cell Signaling), rabbit anti‐p‐Akt Ser473 antibody (1:5000; Abcam), rabbit anti‐iNOS Antibody (1:1000; Cell Signaling) or rabbit anti‐β‐tubulin antibody (1:4000; Cell Signaling).

### 2.8 Immunofluorescence assay

INS‐1 cells were cultured as described above and then seeded onto polylysine‐coated glass cover slips with the indicated drugs. The cells were then fixed with 4% (wt/vol) paraformaldehyde in PBS for 20 minutes. Immunocytofluorescent staining was performed as described previously.^19^

### 2.9 DNA methylation analyses

Genomic DNA was extracted from INS‐1 cells with a Genomic DNA Purification Kit (Qiagen) following the manufacturer's instructions. Bisulphite modification of DNA was carried out with a CpGenome DNA Modification Kit (S7820; Millipore). The specific steps were in accordance with the manufacturer's instructions. The precipitated DNA was washed and collected for the following DNA methylation analyses. PCR amplification products were gel purified and used for TOPO‐TA cloning (Vazyme Biotech), followed by sequencing. The assay was performed as in 20. The methylation level of each indicated CpG islands in genomic region in each sample was measured as the sum of the methylation level of every CpG site divided by the total number of the CpG sites that we covered in the given region. The sequences were then analysed with the QUMA program (http://quma.cdb.riken.jp/).

### 2.10 Statistical analysis

Data analysis was performed with one‐way ANOVA with the Tukey's post hoc test (multiple groups) or the Student *t* test (two groups). Data are expressed as mean ± SEM. SPSS 22.0 was used for analysis. All results were obtained from at least three independent experiments. A value of *P* < .05 was considered statistically significant, and significance is shown in each Figure.

## 3 RESULTS

### 3.1 IL‐1β impairs insulin secretion function in INS‐1 cells and rat islets

**FIGURE 1:**
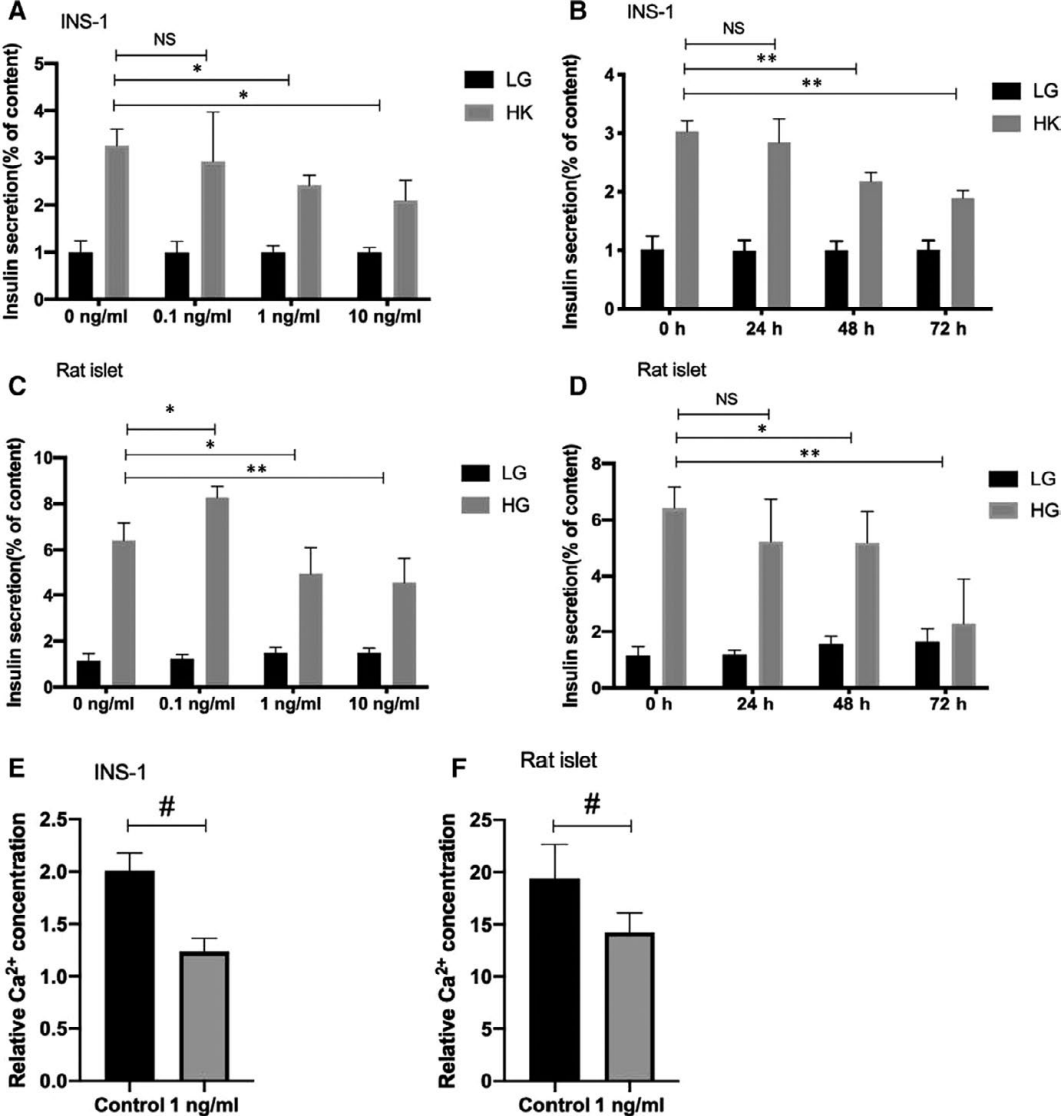
IL‐1β impairs insulin secretion function in INS‐1 cells and rat islets. A, Measurement (% of content) of KSIS from INS‐1 cels treated with IL‐1β (0, 0.1, 1, 10 ng/mL IL‐1β) for 48 h, then in glucose‐free KRB buffer for 1 h, and in KRB buffer with 2.8 mmol/L glucose (LG) or 50 mmol/L K^+^ (HK) for 1 h. B, Measurement (% of content) of KSIS from INS‐1 cells treated with 1 ng/mL IL‐1β for different times (0, 24, 48 or 72 h). C, Measurement (% of content) of GSIS from SD rat islets treated with different concentration (0, 0.1, 1, 10 ng/mL IL‐1β) for 48 h, then in glucose‐free KRB buffer for 1 h, and in KRB buffer with 3.3 mmol/L glucose (LG) or 16.7 mmol/L glucose (HG) for 1 h. D, Measurement (% of content) of GSIS from SD rat islets treated with 1 ng/mL IL‐1β for different times (0, 24, 48 or 72 h). Insulin secretion (% of content) was normalized to the corresponding total insulin content. Insulin levels were detected by RIA. E, Relative HK (2.8 mmol/L glucose and 50 mmol/L K^+^) induced cytoplasmic Ca^2+^ increase was measured in INS‐1 cells treated with 1 ng/mL IL‐1β for 48 h. F, Relative HG (16.7 mmol/L glucose) induced cytoplasmic Ca^2+^ increase was measured in rat islets treated with 1 ng/mL IL‐1β for 48 h. Data are presented as the mean ± SEM (n = 6) of three independent experiments. *P < .05, **P < .01 vs 0 h HK, #P < .05 vs control, NS means no significance

To assess the specific effect of IL‐1β on insulin secretion function in pancreatic β‐cells, INS‐1 cells and rat islets were used in this study. INS‐1 cells were incubated with different concentration IL‐1β for 48 hours and 1 ng/mL IL‐1β for different time. As is shown in Figure 1A, KSIS corrected with total insulin content decreased markedly when the cells were incubated with 1 ng/mL of IL‐1β for 48 hours. As shown in Figure 1B, when INS‐1 cells were treated with 1 ng/mL IL‐1β for different time, insulin secretion initiated to decline from 48 hours, independent of the subsequent KSIS. The rat islets were gained the same treated condition as INS‐1; the results showed that GSIS function is similar to the damaged KSIS of cells (Figure 1C and D). However, under the condition of 0.1 ng/mL concentration, the insulin secretion function was slightly up‐regulated. Particularly, the total insulin content in INS‐1 cells and rat islets was decreased in a dosage‐dependent manner through the suppression of IL‐1β (Figure S1). The results indicated that IL‐1β not only suppresses the total insulin content in islet β‐cells, but also indeed impairs the insulin secretion function. To further proof that IL‐1β indeed impairs the insulin secretion function, cytoplasmic‐free Ca^2+^ concentration was measured. Data showed that cytoplasmic Ca^2+^ spiking responded to high K^+^ or high glucose was reduced in amplitude with 1 ng/mL of IL‐1β treatment for 48 hours in INS‐1 cells or islets (Figure 1E and F). These data complement our previous work and demonstrate that IL‐1β indeed impairs the insulin secretion function.

### 3.2 IL‐1β treatment suppresses *Cask* expression activity in INS‐1 cells and rat islets

**FIGURE 2:**
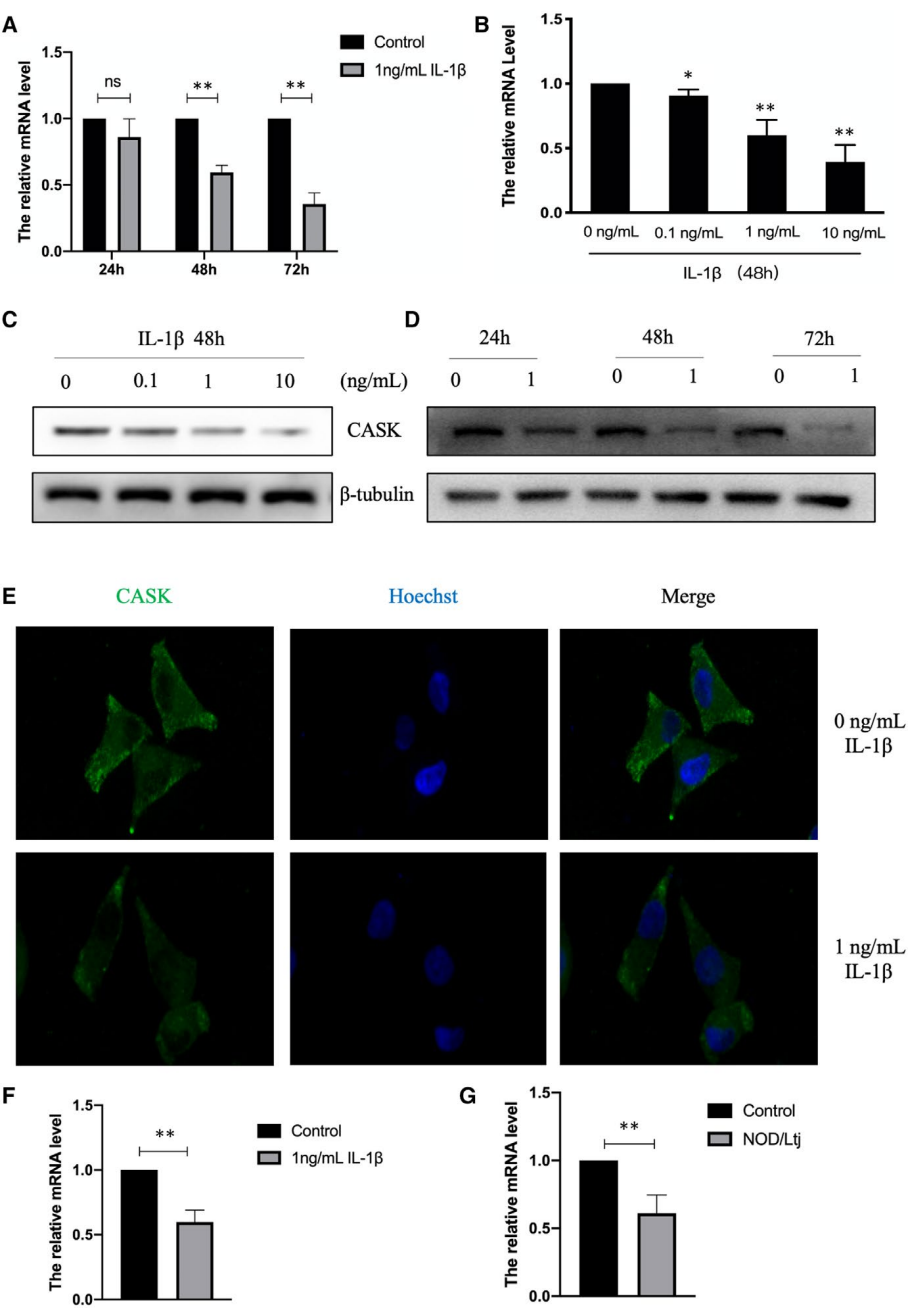
IL‐1β treatment suppresses *Cask* expression activity in INS‐1 cells and rat islets. The expression of *Cask* was analysed by qPCR from INS‐1 cells treated with 1 ng/mL IL‐1β for different times (24, 48 or 72 h) (A) or with various concentrations (0, 0.1, 1, and 10 ng/mL) of IL‐1β for 48 h (B). (C and D) The protein levels of CASK were analysed by Western blot. β‐Tubulin was used as the internal control. (E) The location of CASK was analysed by immunofluorescence assay. The images were recorded at a magnification of ×400. (F) The expression of *Cask* was analysed by qPCR from rat islets treated with 1 ng/mL IL‐1β for 48 h. (G) The expression level of *Cask* was analysed by qPCR islets from NOD/LtJ and control mice without diabetes. Data are presented as the mean ± SEM (n = 3) of three independent experiments. *P < .05, **P < .01 vs control

*Cask* expression, the impact of IL‐1β, was detected using qPCR and Western blotting. INS‐1 cells were exposed under different concentrations of IL‐1β for 48 hours or with IL‐1β (1 ng/mL) for different times. The expression level of *Cask* mRNA was declined to nearly 40% than the control group, with 1 ng/mL IL‐1β for 48 hours (Figure 2A and B), which was verified by Western blotting (Figure 2C and D). Immunofluorescence assay was also used for close observation. The results showed that IL‐1β down‐regulated CASK expression but did not alter its localization in INS‐1 cells (Figure 2E). In SD rat islets, the expression activity of *Cask* was also down‐regulated under 1 ng/mL IL‐1β for 48 hours (Figure 2F). And the expression level of *Cask* was also significantly decreased in the islets of NOD/LtJ mice by comparison with non‐diabetes control mice (Figure 2F).

### 3.3 Up‐regulation of CASK partially restores IL‐1β–induced the dysfunction of insulin secretion in INS‐1 cells

**FIGURE 3:**
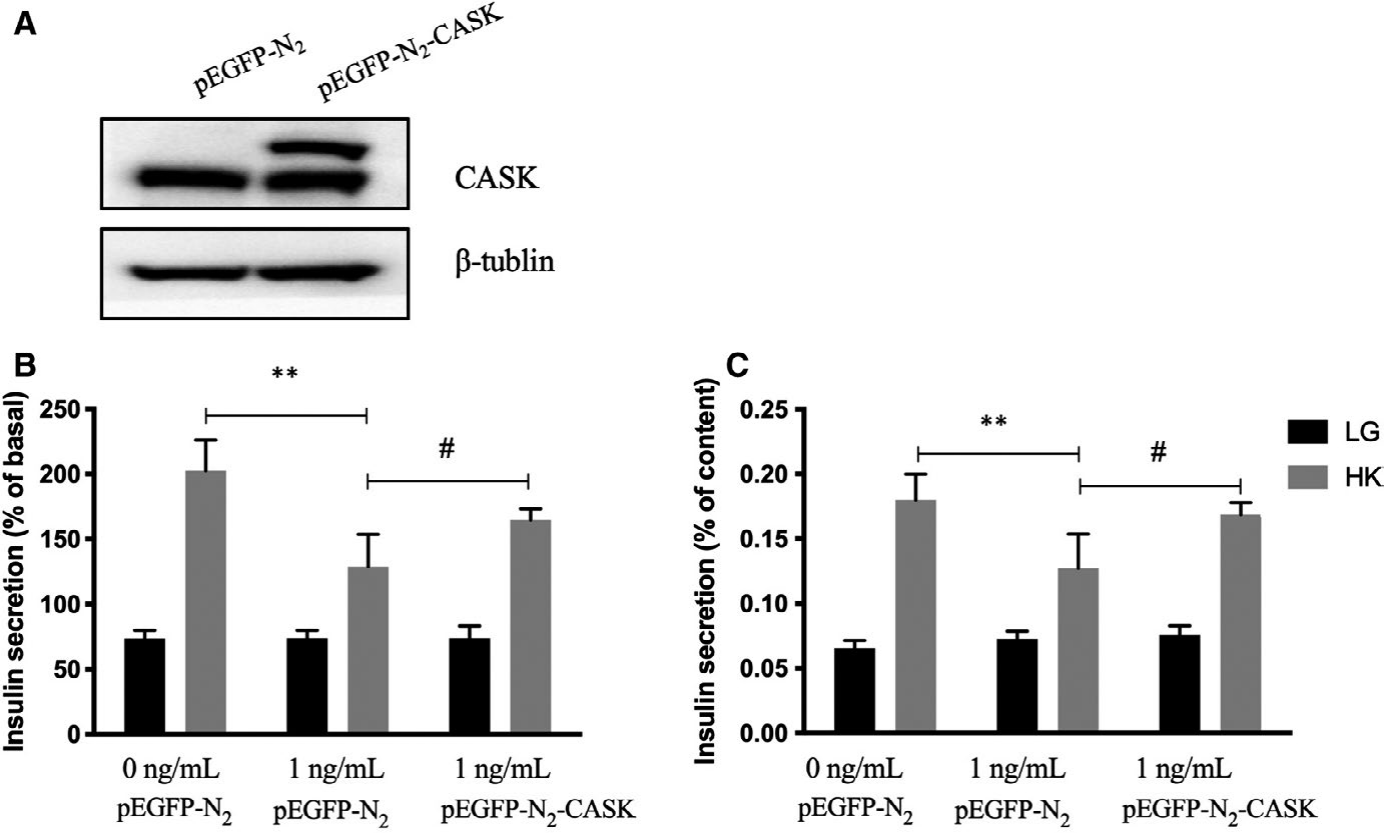
Up‐regulation of CASK partially restores IL‐1β–induced the dysfunction of insulin secretion in INS‐1 cells. A, INS‐1 cells were transfected with CASK overexpression plasmid (pEGFP‐N_2_‐CASK) or control plasmid (pEGFP‐N_2_) for 48 h. The expression level of CASK was analysed by Western blot. β‐Tubulin was used as the internal control. B and C, INS‐1 cells were transfected with CASK overexpression plasmid or control plasmid for 48 h with or without 1 ng/mL IL‐1β, then cultured in glucose‐free KRB buffer for 1 h, and in KRB buffer with 2.8 mmol/L glucose (LG) or 50 mmol/L K^+^ (HK) for 1 h. Insulin levels were detected by RIA. Data are presented as the mean ± SEM (n = 6) of three independent experiments. **P < .01, #*P* < .01, NS means no significance

The CASK protein levels were confirmed by Western blots to determine whether CASK overexpression plasmid (pEGFP‐N_2_‐CASK) could effectively up‐regulate the expression (Figure 3A). To further investigate the part of CASK in insulin secretion defects caused by IL‐1β, the CASK was overexpressed in INS‐1 cells. At the same time, the cells were incubated with 1 ng/mL IL‐1β for 48 hours; the decreased KSIS caused by IL‐1β was partially reversed (Figure 1B and C). These results revealed that CASK is involved in this pathological process that IL‐1β induced the detriment effect of insulin secretion under the stimulation of high potassium in INS‐1 cells.

### 3.4 Activation of DNA methyltransferases was induced by IL‐1β via iNOS

**FIGURE 4:**
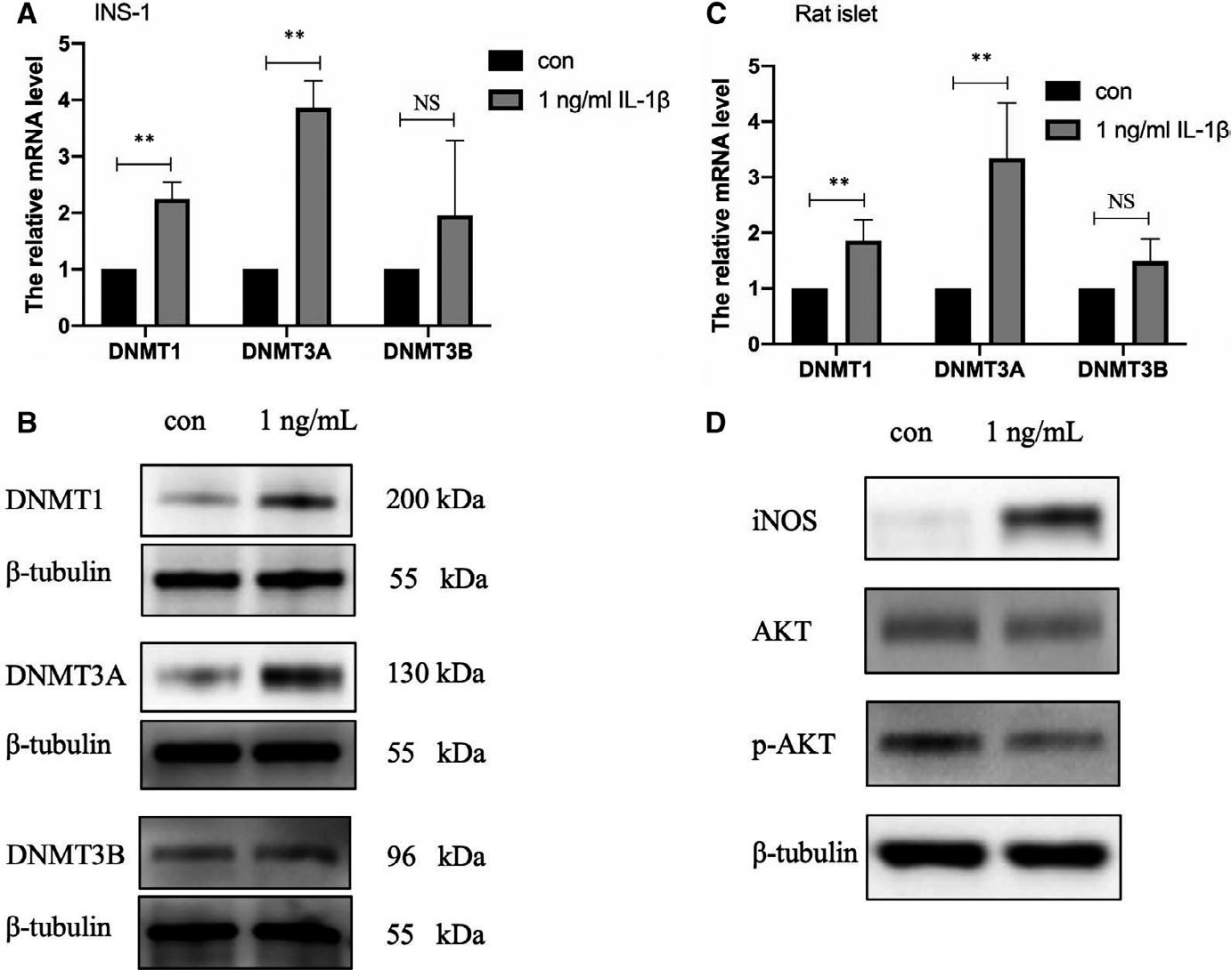
Activation of DNA methyltransferases was induced by IL‐1β via iNOS. INS‐1 cells were treated with 1 ng/mL IL‐1β for 48 h. For DNMT3a, DNMT3b and DNMT1, the mRNA levels and protein levels were analysed by real‐time PCR (A) and Western blot analysis (B), respectively. β‐Tubulin was used as the internal control. (C) The mRNA levels of these DNMT were detected in rat islets treated with 1 ng/mL IL‐1β for 48 h. (D) The expression level of iNOS, Akt, p‐Akt was detected by Western blot analysis. Data are presented as the mean ± SEM (n = 3) of three independent experiments. *P < .05, **P < .01

To investigate DNA methylation modification under IL‐1β exposure in islet β‐cells, we detected the activity of 3 DNA methyltransferases. Western blot and qRT‐PCR assays were carried out to detect the expression of three kinds of DNA methyltransferases—DNMT3a, DNMT3b and DNMT1—based upon administration of 1 ng/mL IL‐1β after 48 hours in INS‐1 cells. As is shown in Figure 4A, the mRNA level of DNMT1 was approximately twofold up‐regulated under 1 ng/mL IL‐1β for 48 hours; DNMT3a and DNMT3b increased by approximately 3 and 1.9 times, respectively. Moreover, the protein levels of the three DNA methyltransferases were obviously increased (Figure 4B). In islets, DNMT1 and DNMT3a were significantly elevated, but the increase of DNMT3b was not statistically significant (Figure 4C). To further validate the regulatory mechanisms, the activity of iNOS and Akt was detected here. The data showed that iNOS was significantly activated by IL‐1β (Figure 4D). But, p‐Akt, the active form of Akt, was abated. These results showed that methyltransferase activity is provoked in islet cells under IL‐1β, which would repress the expression of functional genes that are easily modified by methylation, thus affecting the function of islet β‐cells. Furthermore, the effects of IL‐1β on methylation modification appear to be exerted through activation of iNOS, but not Akt.

### 3.5 The negative modulation of *Cask* gene expression by DNA methyltransferase with IL‐1β stimulation

**FIGURE 5:**
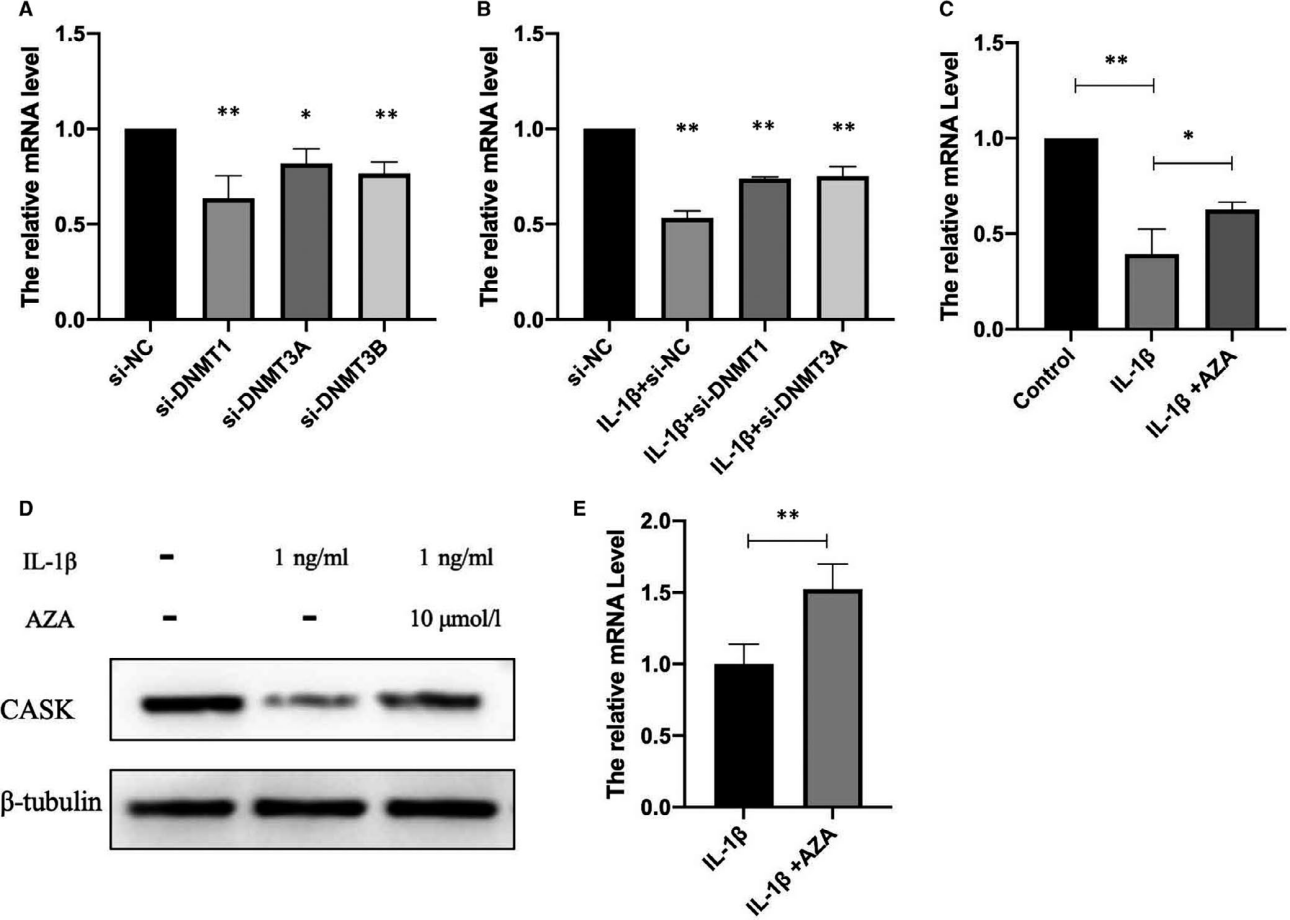
The negative modulation of *Cask* gene expression by DNA methyltransferase with IL‐1β stimulation. INS‐1 cells were respectively transfected with si‐NC, si‐DNMT1, si‐DNMT3A and si‐DNMT3B with or without 1 ng/mL IL‐1β for 48 h. *Cask* mRNA levels in INS‐1 were analysed by real‐time PCR (A,B) (*P < .05, **P < .01 vs si‐NC). INS‐1 cells were cultured with 5‐Aza‐dC and with or without 1 ng/mL IL‐1β for 48 h. 5‐Aza‐dC was added 30 min before IL‐1β treatment. *Cask* mRNA levels in INS‐1 (C) and rat islets (E) were analysed by real‐time PCR, and the protein levels were analysed by Western blot analysis (D), respectively. β‐Tubulin was used as the internal control. Data are presented as the mean ± SEM (n = 3) of three independent experiments (**P < .01 vs. control, *P < .05 vs 50 ng/mL IL‐1β)

To explore whether the negative modulation in CASK by IL‐1β is caused by DNA methyltransferases, si‐DNMTs and methyltransferase non‐selective inhibitor (5‐Aza‐dC) was used.^21^ INS‐1 cells or rat islets were cultured in RPMI‐1640 medium with 5‐Aza‐dC, with or without 1 ng/mL IL‐1β, for 48 hours. The data showed that *Cask* transcription levels were affected and down‐regulated after interfering with the three DNMTs (Figure 5A). Interference with the two increased DNMTs, DNMT1 and DNMT3a, could partially reverse the down‐regulated *Cask* expression level under IL‐1 treatment (Figure 5B). The data also showed that *Cask* mRNA and protein level were partially reversed when INS‐1 cells were incubated with 10 μmol/L 5‐Aza‐dC for 48 hours (Figure 5C and D). The mRNA expression level change was verified in islets (Figure 5C and D). These indicate that the decrease of *Cask* expression caused by IL‐1β is related to the increase of methyltransferase activity.

### 3.6 Insulin secretion function impaired by IL‐1β could be rescued by non‐specific methyltransferase inhibitor 5‐Aza‐dC

**FIGURE 6:**
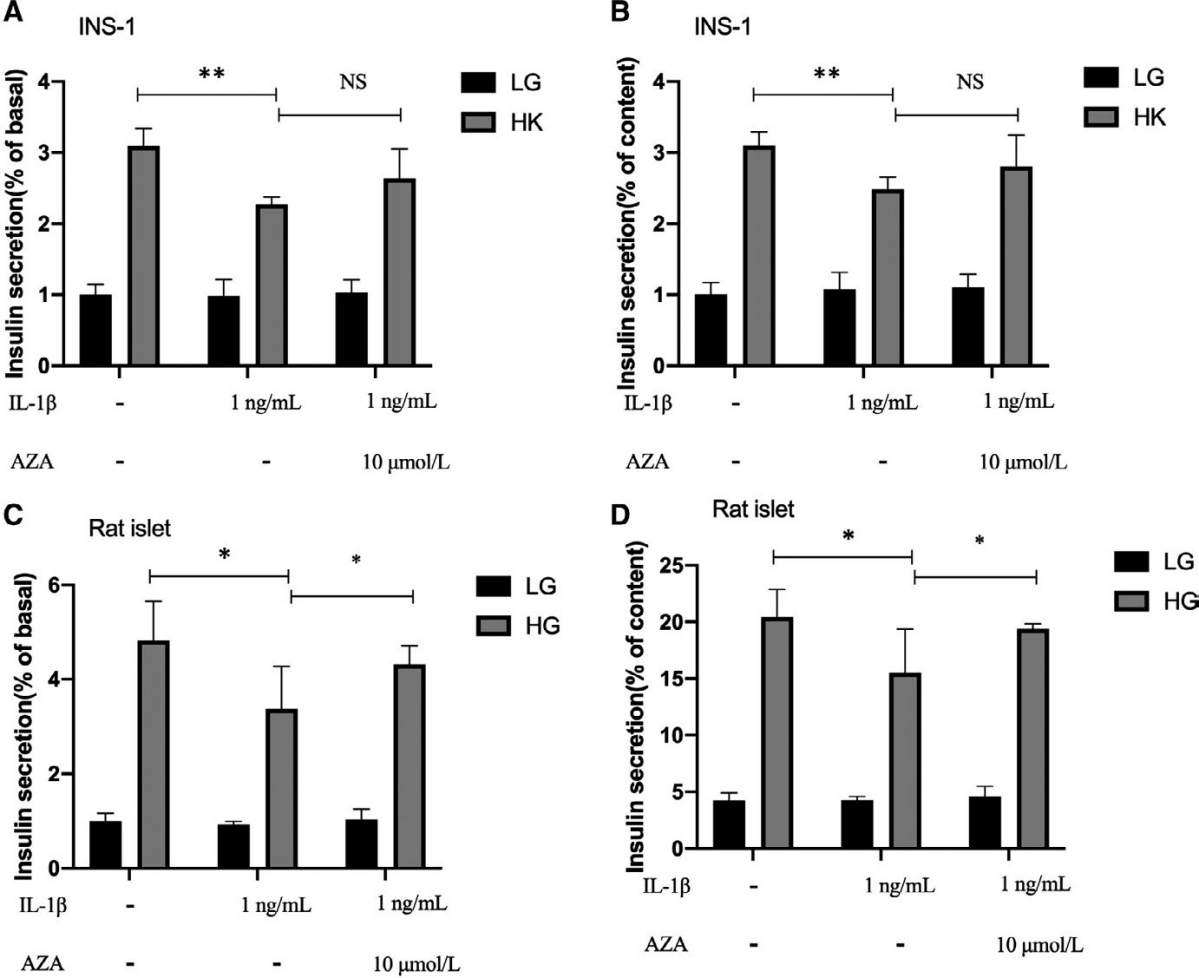
Insulin secretion function impaired by IL‐1β could be rescued by non‐specific methyltransferase inhibitor 5‐Aza‐dC. Measurement (% of basal) and (% of content) of KSIS from INS‐1 cells (A,B) and GSIS from rat islets (C,D) treated with 1 ng/mL IL‐1β for 48 h, 10 μmol/L 5‐Aza‐dC, or both, and then incubated in the conditions described above. Insulin levels were detected by RIA. **P < .01, *P < .05, NS means no significance

To investigate whether insulin secretion defects caused by IL‐1β were related to the increase of methylation activity, we examined KSIS function in INS‐1 cells cultured with 1 ng/mL IL‐1β for 48 hours, 5‐Aza‐dC or both. Insulin secretion ability was slightly up‐regulated by 5‐Aza‐dC (Figure 6A and B). Yet, in islets, GSIS was obviously up‐regulated by 5‐Aza‐dC (Figure 6C and D). The results suggest that IL‐1β–induced insulin secretion damage by DNA methylation.

### 3.7 *Cask* gene promoter hypermethylation in IL‐1β–treated INS‐1 cell

**FIGURE 7:**
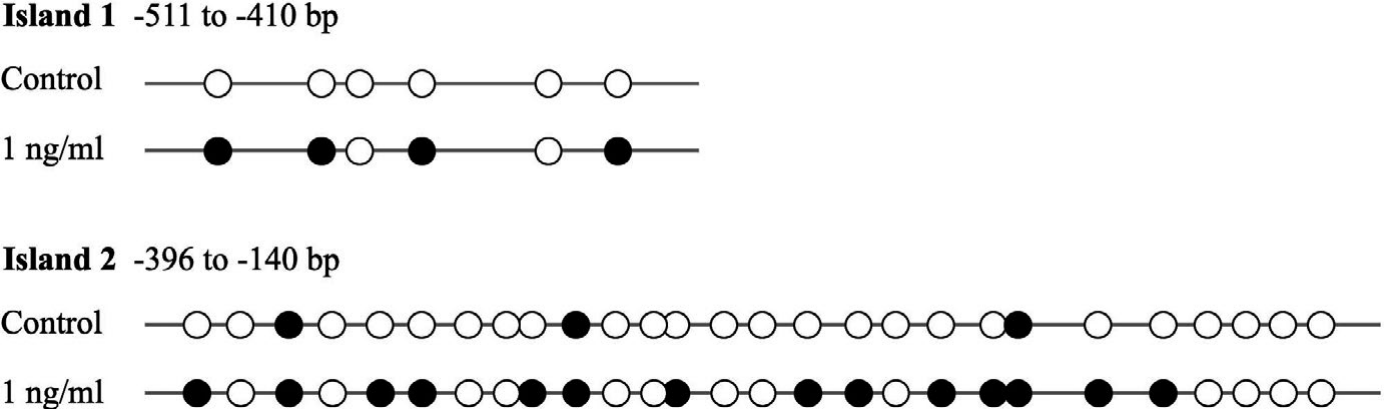
*Cask* gene promoter hypermethylation in IL‐1β–treated INS‐1 cell. Bisulphite sequencing analysis for indicated CpG islands in *Cask* promoter region comparing INS‐1 cells from 1 ng/mL IL‐1β for 48 h treatment and control group (representative result shown here). Each horizontal line with dots is an independent result, and a representative is shown here. Filled black circles represent methylated CpG sites, whereas open circles represent unmethylated CpG sites

Our results clearly suggest that the *Cask* gene may be modified by methylation in this study. To address the mechanism, we assessed whether DNA methylation was involved in the repression of *Cask* genes. The online software EMBOSS Cpgplot was used to predict the CpG islands in the *Cask* gene promoter region (−2000 bp to +50 bp). The results are shown in Figure S2. The genome was extracted from INS‐1 cells treated with 1 ng/mL IL‐1β for 48 hours and modified with bisulphite. Bisulphite sequencing analysis was carried out to confirm methylation level in the CpG‐rich regions of *Cask* gene promotor region. We found that the 2 CpG‐rich regions in the *Cask* promotor (island 1: −511 to −410 bp, island 2: −396 to −140 bp) loci were predominantly methylated in INS‐1 cells under 1 ng/mL IL‐1β for 48 hours compared with control (Figure 7). Moreover, bisulphite sequencing data were quantified and the methylation rate under IL‐1β treatment elevated by about 46% compared with the control group.

## 4 DISCUSSION

Generally, our studies indicate that less CASK expression mediates IL‐1β–induced insulin secretion dysfunction, providing a mechanism for linking chronic islet inflammation to DM. In INS‐1 cells and rat islets, the pathogenic effect of long‐term IL‐1β treatment may promote hypermethylation of the *Cask* promoter region and mediate insufficient levels of CASK. Rectifying the anomalous methylation level of *Cask* partly attenuated this pathogenic effect, which means the gene's vital roles in IL‐1β–induced islet β‐cell dysfunction.

Glucose homeostasis requires sufficient mass of normal‐functioning islet β‐cells.^22^ Recently accumulated evidence manifests that chronic islet inflammation plays an important role in β‐cell dysfunction and failure in diabetes. The relevance between chronic activation of the innate immune system and diabetes has been recognized and has recently gained significant attention owing to elevating evidence of its importance. It has been reported that IL‐1β—the major inflammatory mediator—is elevated in the pancreatic islets of diabetes patients.^2^, ^23^ Plenty of evidence from previous studies suggests that IL‐1β inhibits islet β‐cell proliferation and induces apoptosis, which involves signalling by JNK,^24^ NF‐κB ^25^, ^26^ and the Fas/Fas ligand pathway.^27^, ^28^ Moreover, many studies have indicated that IL‐1β impairs insulin synthesis and decreases islet insulin content.^29^ Commonly, the appropriate insulin level for maintaining glucose homeostasis is determined by normal insulin synthesis and proper insulin secretion. Lowe Jr et al demonstrated that IL‐1β suppresses insulin secretion as the exposure dose increases to 5 ng/mL.^30^ Our results also showed that IL‐1β not only reduces total insulin content (Figure S1) but also contributes to impairment of the insulin secretion function (Figure 1). Moreover, our data showed that cytoplasmic Ca^2+^ spiking responded to high K^+^ or high glucose was reduced in amplitude under 1 ng/mL IL‐1β treatment for 48 hours (Figure 1E and F). It complements our previous work and demonstrates that IL‐1β indeed impairs the insulin secretion function. Although short‐term low‐dose IL‐1β motivates insulin secretion, which consistent with the physiological effect,^31^, ^32^ chronic relatively high concentration stimulation suppressed insulin secretion. All in all, chronic IL‐1β stimulation indeed leads to islet function damage, but the specific modulatory mechanisms of β‐cell dysfunction—especially secretion dysfunction—are still not completely understood.

In mammals, CASK is an essential protein, and CASK^KO^ mice die shortly after birth.^33^ In our previous studies, CASK was found to be a regulator that participates in lipotoxicity, glucotoxicity and Exendin‐4, which affect insulin secretion. We also demonstrated that CASK could reduce the obstructing effect of F‐actin during insulin granule exocytosis and that it is not responsible in the process of energy metabolism and insulin synthesis. Thus, CASK—a cytoskeleton protein is abundant in pancreatic islet β‐cells—is involved in the process of insulin exocytosis.^10^ In the current study, IL‐1β down‐regulated the expression of CASK and impaired islet insulin secretion. Surprisingly, the overexpression of CASK was shown to up‐regulate insulin secretion suppressed by IL‐1β. These findings indicate β‐cell insulin secretion was regulated by a highly complex set of regulatory networks and illuminate the association between them. Consequently, CASK is important for the β‐cell dysfunction induced by chronic inflammation derived from inflammatory factor IL‐1β. The exact mechanism by which CASK regulates and exerts its effects on the insulin secretion process in this study is a key question. Studies have found that CASK is a long‐lived protein that could be regulated in multiple ways, including pro‐translational and post‐translational modification. Translation factors Hif1‐α and FOXO1 could directly regulate *Cask* gene transcription and influence the expression.^10^, ^19^ Moreover, CASK activity and degradation is mediated through the ubiquitin‐proteasome pathway,^34^, ^35^ phosphorylation^36^ and the proteasome‐dependent degradation pathway.^37^ However, it is unclear in which way CASK is modulated in mediating β‐cell damage, and particularly in impairing insulin secretion induced by IL‐1β in this study. Here, our results indicate that the decreased level of expression of CASK was from exposure to IL‐1β, accompanied by the elevation of DNA methyltransferases.

We then focused on the connection between CASK and DNA methyltransferases. DNA methylation at CpG sites in gene promoters—a fundamental epigenetic mechanism—involves direct chemical modification of the DNA, which influences gene activities, regulates protein levels and ultimately shapes phenotypes.^38^ DNA methylation is catalysed by a family of DNA methyltransferases (DNMTs) that includes DNMT3a, DNMT3b and DNMT1.^39^ Accumulated studies have revealed that post‐translational modifications mediated by DNA methylation exert vital effects on pancreatic β‐cell function.^40^, ^41^ More recently, differences have been found between patients with T2DM and non‐diabetic donors in the methylation pattern of pancreatic β‐cells in promoter regions in pancreatic islets, which contributes to perturbing some key functional genes.^42^ On the basis of previous investigations, we explored whether CASK is modified by the specific epigenetic methylation modification method in this study—a major epigenetic factor influencing gene activities. Our bioinformatics analysis revealed that many CpG islands exist in the *Cask* gene promoter region. Consistent with our prediction, the *Cask* promoter was found to be in a hypermethylation state under IL‐1β treatment. After inhibiting the methyltransferase activity by non‐selective methyltransferase inhibitors and si‐DNMTs, the decreasing expression level of CASK was partially reversed. And we have evidence that DNMT can directly affect the case promoter methylation (data not shown). The deficiency of KSIS was also slightly up‐regulated when INS‐1 cells cultured with IL‐1β were incubated with this inhibitor. Distinctly, in islets, the inhibitor could obviously reverse the decreased function of GSIS caused by IL‐1β. KSIS is different from GSIS in that KSIS directly drives the movement of insulin vesicles and promotes insulin secretion by closing the ATP‐sensitive potassium channels. Currently, our results demonstrated that the insulin secretion function defects in islet β cells induced by inflammatory factor IL‐1β are associated with the methylation modification of the *Cask* gene. IL‐1β is known to affect gene expression based on activation of DNMTs in various cell types, but the regulatory mechanism is not clear here. There are two mainly pathways Akt and iNOS involved in the regulation of DNMT activity under IL‐1β.^43^, ^44^, ^45^ To further validate, we detected the activity of iNOS and Akt in current study when expression of *Cask* gene decreased and insulin secretion function was impaired. Our data showed that iNOS was significantly activated by IL‐1β. Yet p‐Akt, the active form of Akt, was abated. Generally, we preliminarily concluded that IL‐1β exposure in islet β cells induces the suppression of the expression of CASK, which could be mediated with activation of DNMTs via iNOS pathway.

In conclusion, our study suggests that CASK forms a part of IL‐1β–inducing insulin secretion defects, which could be mainly exerted through activation of DNA methyltransferase via iNOS. Importantly, our results are the first time to explore the way in which the kinase CASK participates in the process of insulin secretion defects from the perspective of methylation modification. This study broadens our understanding and brings new perspectives on islet inflammation in the development of diabetes.

## CONFLICT OF INTEREST

The authors confirm that there are no conflicts of interest.

## AUTHOR CONTRIBUTIONS

**Tianyuan Wang:** Conceptualization (lead); Writing‐original draft (lead). **Xing‐jing Liu:** Methodology (equal). **Jin‐yang Xie:** Data curation (equal). **Qing‐zhao Yuan:** Formal analysis (equal). **Yao Wang:** Funding acquisition (lead); Resources (lead).

## Supporting information

Table S1‐Fig S1‐S2

## Data Availability

The data used to support the findings of this study are available from the corresponding authors upon request.
