# Supplementary material for: Cask methylation involved in the injury of insulin secretion function caused by interleukin1‐β
Source: J Cell Mol Med. 2020 Nov 14;24(24):14247–56. doi: 10.1111/jcmm.16041 (PMC7753871; doi:10.1111/jcmm.16041)
Supplement: Supplementary file 1 — Table S1‐Fig S1‐S2 [file JCMM-24-14247-s001.docx]

**Supplement Table 1.** **The sequences of qPCR primers**

| **Primers** | **Sequence（5'-3'）** |
| --- | --- |
| Actin-F | TGAACGGGAAGCTCACTGG |
| Actin-R | TCCACCACCCTGTTGCTGTA |
| CASK-F | AAGGAGAAAACTAAAGGGTGC |
| CASK-R | GGAGGTAGGGTCTTCGGAG |
| DNMT1-F | AAGAATGGTGTTGTCTACCGAC |
| DNMT1-R | CATCCAGGTTGCTCCCCTTG |
| DNMT3a-F | GATGAGCCTGAGTATGAGGATGG |
| DNMT3a-R | CAAGACACAATTCGGCCTGG |
| DNMT3b-F | CGTTAATGGGAACTTCAGTGACC |
| DNMT3b-R | CTGCGTGTAATTCAGAAGGCT |

**Supplementary Figure.1**

**
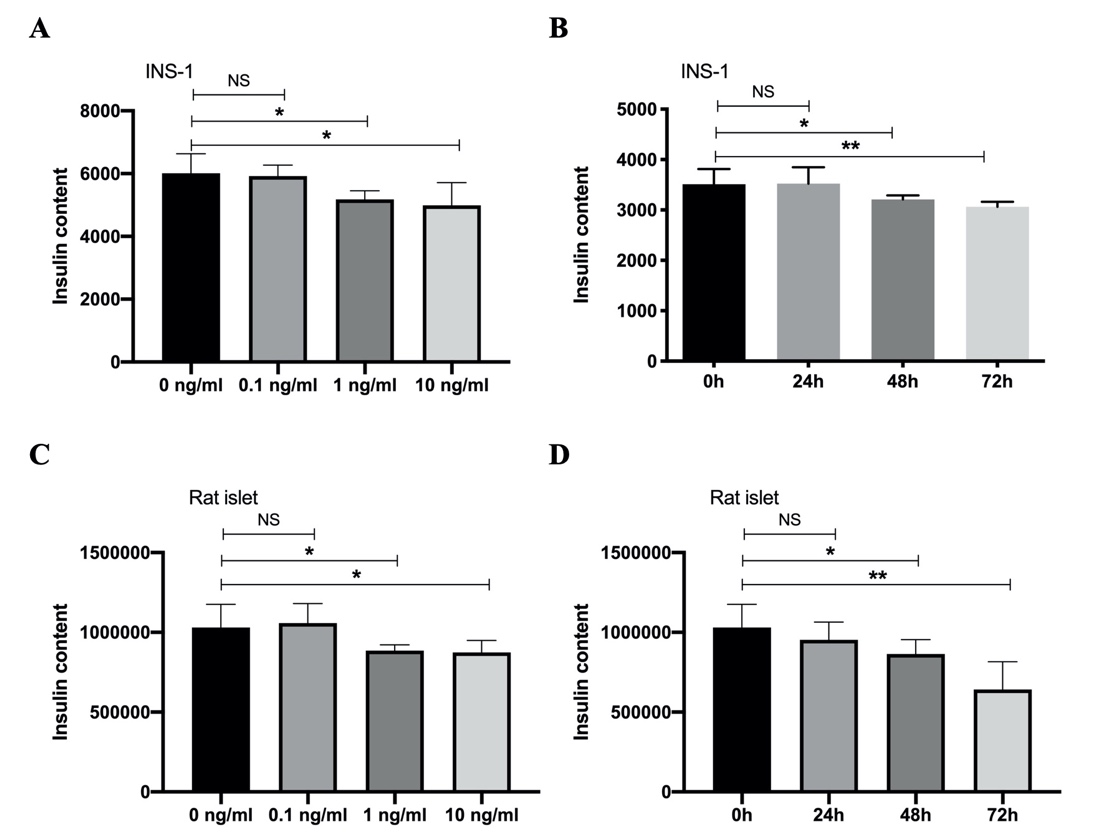
**

**Supplementary Fig.1. IL-1β impairs insulin content in INS-1 cells and rat islets.**

(A) INS-1 cells treated with IL-1β (0, 0.1, 1, 10 ng/mL IL-1β) for 48h, after KSIS assay, the total insulin content of the cells was detected by RIA. (B) INS-1 cells were treated with 1 ng/mL IL-1β for different times (0h, 24h, 48h, or 72h). (C&D) SD rat islets treated with different concentration (0, 0.1, 1, 10 ng/mL IL-1β) for 48h and with 1 ng/mL IL-1β for different times (0h, 24h, 48h, or 72h). Insulin levels were detected by RIA. Data is presented as the mean ± SEM (n = 6) of three independent experiments. *P < 0.05, **P < 0.01 *vs.* control, NS means no significance.

**Supplementary Figure.2**


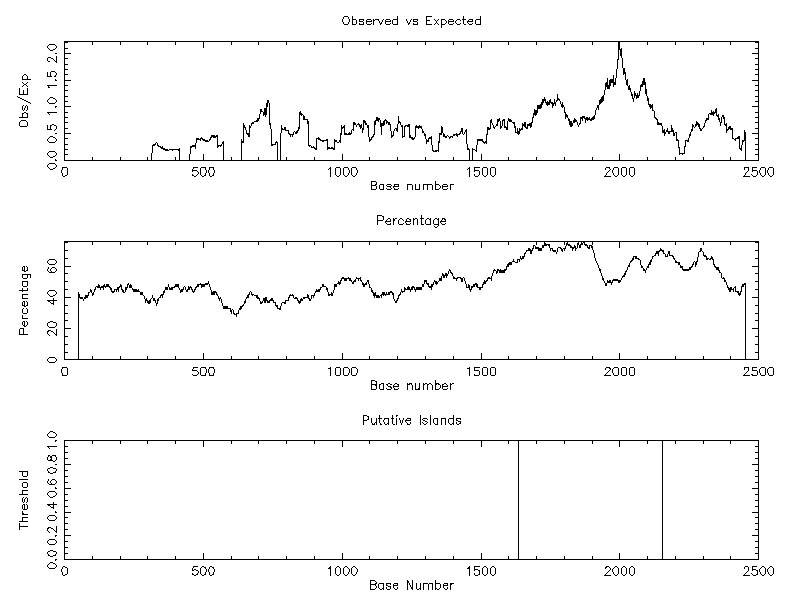


A

B

C

**Supplementary Fig. 2 The prediction of CpG islands in the *Cask* gene promoter region.** This is the result downloaded from online software EMBOSS Cpgplot to predict the CpG islands. A&B. The abscissa refers to the *Cask* gene promoter region (−2000bp to +50bp), the value of the ordinate represents the possibility of CpG island; C. It represents the high GC region, which is the most likely region for methylation.
